# Unravelling the RNA-Binding Properties of SAFB Proteins in Breast Cancer Cells

**DOI:** 10.1155/2015/395816

**Published:** 2015-07-26

**Authors:** Elaine Hong, Andrew Best, Hannah Gautrey, Jas Chin, Anshuli Razdan, Tomaz Curk, David J. Elliott, Alison J. Tyson-Capper

**Affiliations:** ^1^Institute of Cellular Medicine, Faculty of Medical Sciences, Newcastle University, Framlington Place, Newcastle upon Tyne NE2 4HH, UK; ^2^Institute of Genetic Medicine, Faculty of Medical Sciences, Newcastle University, Central Parkway, Newcastle upon Tyne NE1 3BZ, UK; ^3^Faculty of Computer and Information Science, University of Ljubljana, Traska Cesta 25, 51-1000 Ljubljana, Slovenia

## Abstract

Scaffold attachment factor B1 (SAFB1) and SAFB2 proteins are oestrogen (ER) corepressors that bind to and modulate ER activity through chromatin remodelling or interaction with the basal transcription machinery. SAFB proteins also have an internal RNA-recognition motif but little is known about the RNA-binding properties of SAFB1 or SAFB2. We utilised crosslinking and immunoprecipitation (iCLIP) coupled with high-throughput sequencing to enable a transcriptome-wide mapping of SAFB1 protein-RNA interactions in breast cancer MCF-7 cells. Analysis of crosslinking frequency mapped to transcript regions revealed that SAFB1 binds to coding and noncoding RNAs (ncRNAs). The highest proportion of SAFB1 crosslink sites mapped to ncRNAs, followed by intergenic regions, open reading frames (ORFs), introns, and 3′ or 5′ untranslated regions (UTR). Furthermore, we reveal that SAFB1 binds directly to RNA and its binding is particularly enriched at purine-rich sequences not dissimilar to the RNA-binding motifs for SR proteins. Using RNAi, we also show, for the first time, that single depletion of either SAFB1 or SAFB2 leads to an increase in expression of the other SAFB protein in both MCF-7 and MDA-MD231 breast cancer cells.

## 1. Introduction

The growing interest in SAFB1 and SAFB2 proteins in relation to cancer is generated from their well described ability to bind to and modulate ER-*α*, a central player in breast cancer development. Moreover, a role for SAFB1 in RNA splicing and metabolism has also been proposed. Nayler et al. [1] first described interactions between SAFB1 with RNA polymerase II and a subset of serine/arginine-rich RNA processing factors (SR proteins) suggesting that SAFB1 serves as a molecular base for the assembly of a transcriptome complex that couples chromatin organising S/MARs elements with transcription and pre-mRNA processing [1]. Protein-protein interactions between SAFB1 and a range of RNA-binding proteins including hnRNP A1, hnRNP D, hnRNP G, SR splicing regulatory protein 86 (SRrp86), SR protein kinase 1 (SRPK1), and Src-associated substrate in mitosis of 68 kDa (Sam68) provide reasonable evidence to implicate a role in alternative splicing [2–6]. However, it is still not known whether these SAFB proteins exert their effects on pre-mRNA splicing through direct RNA interaction or by tethering to other splicing factors.

SAFB1 and SAFB2 proteins share a highly conserved RNA-recognition motif (RRM) with 98% similarity in the central region, although until now their direct RNA-binding potential has remained unclear. SAFB1 has also been labelled as a novel hnRNP protein due to its similarity to the highly conserved RBD found in the hnRNP protein family [6]. Subsequent studies have implicated both SAFB proteins in alternative splicing, as overexpression of SAFB1 and SAFB2 inhibits splicing of a* TRA2B* variable exon [5, 7]. However, further investigation using mutants lacking the RRM domain revealed that SAFB1's ability to inhibit* TRA2B* exon skipping was independent of its RNA-binding ability [7]. This evidence suggests that SAFB1 may not bind directly to* TRA2B* pre-mRNA to regulate exon skipping but could possibly mediate an indirect effect through its interaction with various splicing factors [2, 4–6]. In an unrelated study,* in vitro *evidence has shown that the RRM domain of SAFB1 was able to bind RNA isolated from MCF-7 breast cancer cells, although the identity of the RNA targets was not described [8]. The current study was designed to establish whether SAFB proteins exert their RNA processing functions through direct RNA interaction as well as by tethering to other protein factors.

## 2. Material and Methods

### 2.1. iCLIP

CLIP with individual nucleotide resolution (iCLIP) was performed for SAFB1 using MCF-7 breast cancer cells based on a published protocol [9]. In brief, MCF-7 cells were irradiated with 150 mJ/cm^2^ of UV at 254 nm and cell pellets resuspended in lysis buffer treated with Turbo DNase I (Ambion) and high (1 : 10 dilution) or low (1 : 500 dilution) RNase I (Ambion). Dynabeads Protein A or Dynabeads Protein G (Invitrogen) were resuspended in lysis buffer containing 5 *μ*g SAFB1 antibody (Sigma-Aldrich) and precleared lysate was added to the magnetic beads for immunoprecipitation at 4°C for 2 hours. RNA 3′ ends were dephosphorylated and RNA linkers ligated. Magnetic beads were then resuspended in PNK mix containing ^32^P-*γ*-ATP to radioactively label the RNA 5′ ends, as previously described [9]. Protein-RNA complexes were isolated following electrophoresis (see Supplementary Figure  1 in Supplementary Material available online at http://dx.doi.org/10.1155/2015/395816). Precipitated RNA was reverse transcribed in RNA/primer mix containing different Rclip primers with individual barcode sequences for each replicate. Three gel fragments corresponding to cDNA size were cut at 120–200 nucleotides (high), 85–120 nucleotides (medium), and 70–85 nucleotides (low) (Supplementary Figure  1(B)). Three independent biological replicates were prepared for sequencing using the TruSeq Sample Preparation kit (Illumina) and sequenced on the Genome Analyser II system (GAIIx, Illumina). Bioinformatic analyses were performed on the web-based iCount software (http://icount.biolab.si/). Mapping of SAFB1 crosslink sites to regions of respective genes was visualised in UCSC Genome Browser (http://genome.ucsc.edu/) and a graphical representation of the novel SAFB1 consensus binding motif was designed using the web-based WebLogo software (http://weblogo.berkeley.edu/). Potential target genes that contain SAFB1 binding sites were selected for further validation using qRT-PCR with TaqMan gene expression assays.

#### 2.1.1. Transient Transfections

MCF-7 and MDA-MB-231 cells were reverse transfected with two independent sets of Silencer Select siRNA for SAFB1 and SAFB2,* Silencer* Negative Control siRNA,* Silencer* Select GAPDH, and *β*-actin Positive Control siRNA (Life Technologies) using INTERFERin Transfection Agent (Polyplus transfection) according to the manufacturer's instructions. Following optimisation, cells were seeded at 3.5 × 10^4^ cells/well in 24-well plates or 1.75 × 10^5^ cells/well into 6-well plates. After 24 hours, cells were transfected with 5 nM siRNA with 4 *μ*L/mL of INTERFERin transfection agent. RNA was collected 48 and 72 hours after transfection and protein collected 72 hours after transfection. In all experiments, levels of knockdown by RNAi were assessed at the RNA and protein level by PCR and immunoblotting.

#### 2.1.2. RNA Isolation and PCR

Total RNA from cultured cells was extracted with RNeasy spin columns (Qiagen) or the SV Total RNA Isolation System (Promega) according to manufacturer's instructions. One *μ*g of total RNA was reverse transcribed using oligo (dT) primers and Superscript II Reverse Transcriptase (RT) (Invitrogen, Life Technologies) following manufacturer's instructions. Conventional PCR was performed using PCR master mix (Promega). qPCR was performed using TaqMan gene expression assays (Life Technologies) according to the manufacturer's instructions. Validated TaqMan probes were selected to target specific genes as follows: SAFB1 (Hs01561652_gl), SAFB2 (Hs01006796_g1), ITGB4 (Hs00236216_m1), SHF (Hs00403125_m1), MALAT-1 (Hs00273907_s1), and *β*-actin (Hs99999903_m1). Data analysis was performed using the comparative C_t_ method normalised against *β*-actin expression. Experiments were performed in triplicate and statistical analysis was performed using Student's *t*-test or Repeated Measures ANOVA with Dunnett's Multiple Comparison Test. All effects at *P* < 0.05 are reported as significant.

### 2.2. RNA Immunoprecipitation

MCF-7 cells were washed in ice cold PBS and then collected in lysis buffer (10 mM Tris-HCl (pH 7.5), 150 mM NaCl, 0.5% NP-40, and 1% Triton X-100) containing protease inhibitors (Sigma-Aldrich) and RNase OUT (Invitrogen). Equal amounts of cell lysate were then incubated for 3 hours at 4°C with 2 *μ*g of either rabbit anti-SAFB1 antibody (Genetex) or rabbit IgG control (Santa Cruz). Cell lysates and antibodies were then incubated with Dynabeads Protein G (Invitrogen) for a further hour at 4°C. Beads were then washed five times with lysis buffer before the immunoprecipitated RNA was collected in Trizol reagent (Invitrogen) according to manufacturer's instructions. The RT-PCR was performed as before but half of the RNA obtained from the immunoprecipitation was used in each reaction. Fold enrichment of target mRNA was determined after normalization to the input and rabbit IgG controls.

## 3. Results

### 3.1. Identification of RNA-Binding Sites for SAFB1 in Breast Cancer Cells

Although the role of SAFB proteins in RNA processing has been speculated, the function of their highly homologous internal RRM has not been examined. We sought to identify possible direct RNA targets for SAFB1 in breast cancer cells, using iCLIP technology [9–12] combined with high-throughput sequencing and mapping to generate a transcriptome-wide binding map for SAFB1. SAFB1 protein-RNA complexes were successfully generated by immunoprecipitation and RNA recovered and purified from 3 independent iCLIP replicates (Supplementary Figure  1). High-throughput sequencing and bioinformatics generated a total of 1,145,271 unique cDNA reads with single-hits mapping to the human genome which were subsequently filtered down to 587,119 significant unique cDNAs distributed over 127308 binding sites in 25207 SAFB1 crosslink clusters (FDR < 0.05). A snapshot of the view for SAFB1 crosslink sites on the UCSC Genome Browser (http://genome.ucsc.edu/) is shown in Supplementary Figure  1(C).

**Figure 1:**
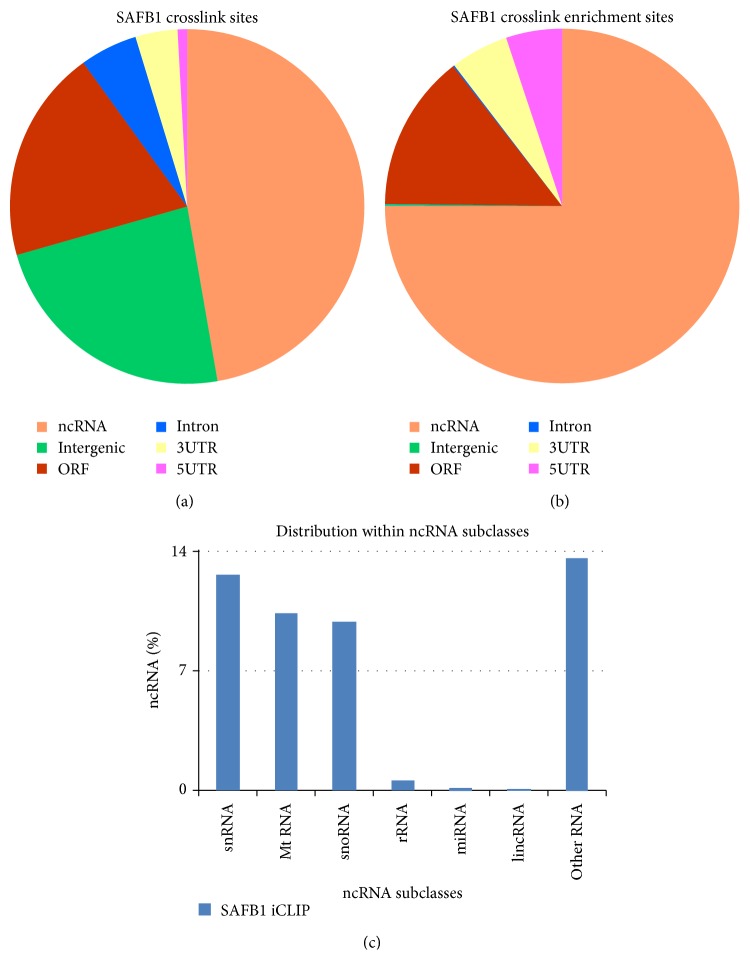
Distribution of significant SAFB1 crosslink sites within RNA segment types. (a) The proportion of cDNAs mapped to different transcript regions relative to the total number of cDNA reads revealed that the highest percentage of cDNAs was mapped to ncRNA (47.08%), followed by intergenic regions (23.24%), ORFs (19.38%), introns (5.23%), 3′ UTRs (3.83%), and 5′ UTRs (0.86%). (b) The fold enrichment of cDNA density in different types of RNAs relative to cDNA density in the whole genome highest density enrichment in ncRNAs. (c) The distribution of SAFB1 crosslink sites within different ncRNA subclasses revealed significant abundance in snRNA, Mt RNA, and snoRNA. “Other RNA” consists of pseudogenes and processed transcripts with no known ORF or function.

iCLIP identified binding sites for SAFB1 across the whole transcriptome, where 100% of significant cDNA reads mapped to the sense orientation in annotated genes. This confirms the high strand specificity of iCLIP also observed in other studies [11, 13]. Analysis of crosslinking frequency mapped to transcript regions revealed that SAFB1 binds to coding and noncoding RNAs (ncRNAs). Notably, the highest proportion of 127308 SAFB1 crosslink sites from significant clusters map to ncRNAs followed by intergenic regions, open reading frames (ORFs), introns, and 3′ or 5′ untranslated regions (UTRs) (Figure 1(a)). When the cDNA density for each transcript region was analysed relative to the cDNA density in the whole genome, the highest density enrichment was detected in ncRNAs (Figure 1(b)). The distribution of SAFB1 crosslink sites within ncRNA subclasses was also analysed. SAFB1 crosslink sites were most abundant in small nuclear RNA (snRNA), mitochondrial RNA (Mt RNA), and small nucleolar RNA (snoRNA) (Figure 1(c)).

### 3.2. Identification of an RNA-Binding Motif for SAFB1

**Figure 2:**
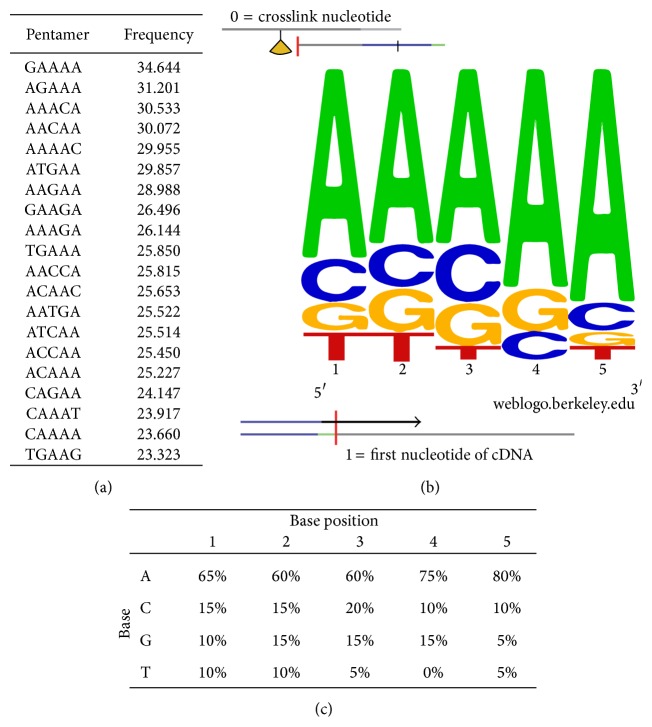
*In vivo* consensus binding motif of SAFB1. (a) The frequency of pentamers surrounding SAFB1 crosslink sites was determined. Adenine represents 68% of the 20 pentamers that has the highest frequencies. (b) WebLogo showing base frequencies of each base at respective positions of the pentamer. SAFB1 binds to adenine-rich motifs. (c) The frequency of each base relative to its position within the pentamer was summarised in this table. The highest frequency of adenine was observed at base position 5; thymine was excluded at base position 4 of the consensus binding motif. This consensus binding motif was predicted from iCLIP cDNA libraries; therefore the uracil base is referred to as thymine in these sequences.

The* in vivo* binding specificity of SAFB1 is still currently unknown. The advantage of single nucleotide resolution provided by iCLIP method enabled the assessment of sequence specificity for SAFB1 binding. To derive whether a consensus binding motif exists for SAFB1, enriched pentamer sequences surrounding the crosslink sites were identified. The frequencies of each pentamer were analysed to determine the top 20 pentamers for SAFB1. Strikingly, adenine appeared as the most frequent nucleotide in the top 20 pentamers and represents 68% of the enriched pentamers (Figure 2(a)). The predicted SAFB1 consensus binding motif contains adenine-rich sequences derived from the pentamers (Figure 2(b)). When the frequency of each nucleotide in the cDNA libraries was analysed relative to its base position, a strong inclusion of adenine at base position 5 was observed (80%) while thymine (uracil in RNA) was excluded at base position 4 of the putative RNA-binding motif (Figure 2(c)). The consensus binding motif for SAFB1 has not been described before; therefore, this novel finding is likely to be of significance to further our current understanding of SAFB1 RNA-binding specificity.

### 3.3. Identification of Novel RNA Targets from Data Generated by iCLIP

**Figure 3:**
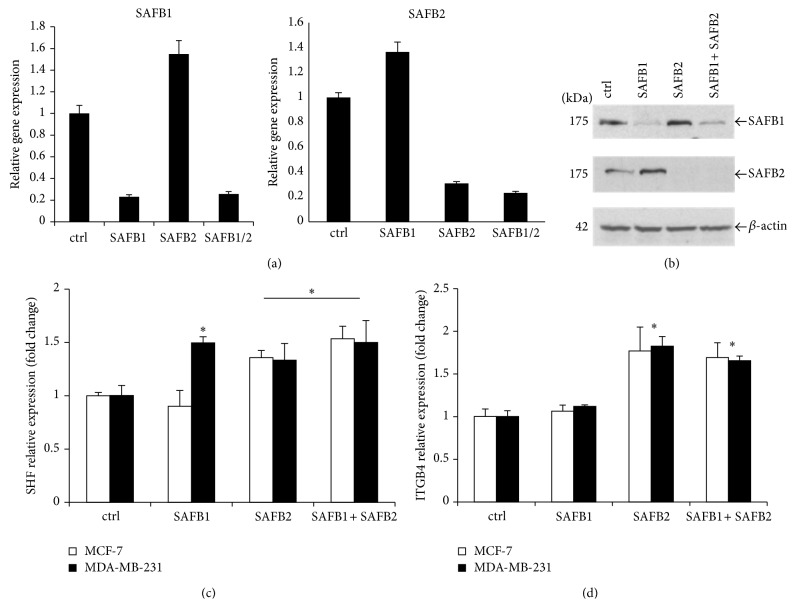
Loss of SAFB proteins affects expression of* SHF* and* ITGB4.* Knockdown of SAFB1 in MCF-7 cells increases mRNA and protein expression of SAFB2 and similarly knockdown of SAFB2 leads to increased expression of SAFB1. MC7-7 cells were transiently transfected with negative, SAFB1, SAFB2 or SAFB1 and SAFB2 siRNA. mRNA levels were measured by qRT-PCR. Data represents the average of three biological replicates ± SD. Statistical significance of mRNA expression was calculated using Student's *t*-test; ^∗^ = *P* < 0.05. (b) Protein levels were analysed by immunoblotting using SAFB1 and SAFB2 antibodies. (c) The effect of SAFB knockdown on* SHF* and* ITGB4* expression by qRT-PCR using validated TaqMan probes specifically targeting* SHF* (c) or* ITGB4* (d). Data represents the average of three biological replicates ± SD. Statistical significance of mRNA expression was calculated using Student's *t*-test; *P* < 0.05.

Data analysis of bound RNAs revealed the number of SAFB1 crosslink sites within each RNA target. When the top 10 RNA targets with the largest number of crosslink sites were listed according to each RNA segment, the position of SAFB1 binding within each gene was visualised using the UCSC Genome Browser (Supplementary Figure  2). This enabled the identification of several interesting RNA targets that were selected for validation. Further experimentations were performed using qRT-PCR or conventional PCR on RNAi transfected MCF-7 and MDA-MB-231 cells to verify the effect of loss of SAFB1 on the expression of these selected RNA targets. Since SAFB2 shares 98% sequence homology to the RRM of SAFB1, these cells were also depleted of SAFB2 and double knockdown of SAFB1 and SAFB2 was also included; interestingly, data shows that when MCF-7 cells are reduced of SAFB1 by RNAi, the levels of SAFB2 mRNA and protein increase (Figure 3(a)). Likewise, levels of SAFB1 increase after knockdown of SAFB2 (Figure 3(b)). A similar pattern was observed when the breast cancer MDA-MB-231 cells were used (Supplementary Figure  3).

Analysis of the RNA map revealed a large number of SAFB1 binding sites on the* SHF* mRNA, particularly accumulated around the alternative promoter (Supplementary Figure  3); the use of alternative promoters plays a significant role in gene expression control (reviewed in [14–16]). More importantly, the aberrant use of alternative promoter has been linked to a number of diseases, including cancer [17]. Therefore, identification of* SHF* as a potential RNA target for SAFB1 warrants further investigation. In MCF-7 noninvasive breast cancer cells, a reduction in SAFB1 did not appear to significantly alter* SHF* mRNA expression, whereas in MDA-MB-231 invasive breast cancer cells there was a significant increase in* SHF* mRNA expression when SAFB1 was reduced (Figure 3(c)). Loss of SAFB2 and both SAFB proteins by RNAi increased* SHF* expression, again supporting their role as transcriptional repressors. Another potential RNA target for SAFB proteins is* ITGB4*. The observed loss of SAFB1 in both breast cancer cell lines does not have an effect on* ITGB4* mRNA expression whereas loss of SAFB2 and both SAFB proteins significantly increased* ITGB4* expression (Figure 3(d)).

### 3.4. Malat-1: A ncRNA Target for SAF2?

**Figure 4:**
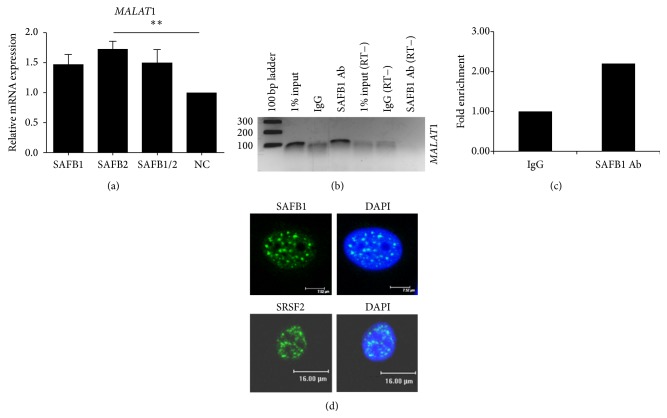
SAFB2 regulates expression of* MALAT-1*. (a) Expression of* MALAT-1* was measured by qRT-PCR using RNA from MCF-7 and MDA-MB-231 cells transfected with negative, SAFB1, SAFB2 or SAFB1 and SAFB2 siRNA using validated TaqMan probes specifically targeting* MALAT-1.* Data represents the average of three biological replicates ± SD. Repeated Measures ANOVA with Dunnett's Multiple Comparison Test statistical significance of mRNA expression was calculated using Student's *t*-test; *P* < 0.01. Enrichment of* MALAT1* RNA by conventional PCR (b) and qPCR (c) after RNA immunoprecipitation with anti-SAFB1 in MCF-7 cells. No enrichment was observed using IgG. (d) Intranuclear distribution of SAFB1 and SRSF2 in MCF-7 cells by immunofluorescent staining. Confocal laser microscopy revealed a punctate pattern for SAFB1 and SRSF2 in nuclear speckles.

Another interesting observation from the iCLIP dataset revealed significant SAFB1 binding sites to metastasis associated lung adenocarcinoma transcript 1 (*MALAT-1*).* MALAT-1* is a highly conserved long ncRNA enriched in nuclear speckles that regulates alternative splicing by modulating splicing factor phosphorylation [18].* MALAT-1* is overexpressed in many different cancers including breast and is considered an oncogenic long ncRNA [19, 20]. We show that, in MCF-7 cells, loss of SAFB2 resulted in an increase in the levels of* MALAT-1* expression (Figure 4(a)). Moreover, we also tested the ability of SAFBI to immunoprecipitate* MALAT-1* RNA in MCF-7 cells; enrichment of* MALAT-1* RNA was observed and determined by conventional PCR and qPCR (Figures 4(b) and 4(c)).

## 4. Discussion

The presence of the highly conserved RRMs within SAFB1 and SAFB2 proteins has been a subject of interest since their discovery, especially in relation to their RNA-binding potential. Despite the fascination, very little has been undertaken until now to describe their RNA-binding capabilities. Initial* in vitro* evidence showed that the RRM of SAFB1 is able to bind RNA when glutathione S-transferase- (GST-) tagged SAFB1 protein combined with total RNA from MCF-7 cells generated a PCR product when reverse transcribed and PCR amplified [8]. However, the identity of the RNA targets was not described and important questions with respect to the role of SAFB1 and SAFB2 in RNA processing remained unanswered.

iCLIP has been proven as a powerful method to determine protein-RNA interactions* in vivo* on a global scale and identify the positions of crosslink sites at nucleotide resolution [11]. The random barcode incorporated to individual cDNA molecules addresses the problem of PCR artifacts faced by all high-throughput sequencing methods. iCLIP has generated a huge dataset and this is an initial analysis of the RNA-binding data for SAFB1. In this study, a global view comparison of the complete dataset from each individual biological replicate showed that all datasets generated consistent and reproducible results, underlining the high quality iCLIP data achieved by high stringency purification and library preparation.

The identification of* in vivo* targets by iCLIP enabled the mapping of transcript regions and RNA classes bound by SAFB1. An overview of the iCLIP results showed that the important class of RNAs bound by SAFB1 was ncRNAs. Interestingly, this binding distribution of SAFB1 is similar to the RNA-binding distribution of splicing factors SRSF3 and SRSF4 rather than hnRNP C protein; SAFB1 was initially classified as a novel member of the hnRNP protein family [6]. Recent work by Änkö et al. utilised iCLIP to reveal that concentrated SRSF3 and SRSF4 binding sites were also in ncRNAs [21], while König et al. [11] showed that hnRNP C binding sites were most abundant within introns [11]. This observation raises the possibility that SAFB1 protein may have similar characteristics to SR proteins rather than hnRNP protein members, although at this stage this is only speculative.

The term ncRNA is commonly used for RNA that does not encode a protein but appears to comprise internal signals that control various levels of gene expression, including chromatin organisation, transcription, RNA splicing, editing, translation, and turnover (reviewed in [22]). Consistent with already known functions of SAFB proteins, concentrated SAFB1 binding in ncRNAs observed from the iCLIP data could possibly contribute to its various role in chromatin organisation, transcription, and RNA metabolism. Analysis of SAFB1 distribution within ncRNA subclasses revealed most abundant SAFB1 binding in snRNAs. snRNAs are a class of small RNA molecules found to be uridylate-rich and localised within the nucleus [23]. The most common members of snRNAs are the U1, U2, U4, U5, and U6 snRNAs that form the spliceosome along with many other protein factors and primarily function in pre-mRNA splicing (reviewed in [24]). The high distribution of SAFB1 binding sites in snRNAs observed in this study supports previously identified interactions between SAFB1 with various RNA processing factors and splicing machinery [1, 4, 6, 25].

The genome-wide, single nucleotide resolution of iCLIP data enabled the prediction of* in vivo* consensus binding sequences for SAFB1 based on the enriched pentamer sequences surrounding the crosslink sites. This study is the first to report that SAFB1 binds a consensus adenine-rich sequence* in vivo.* Closer examination of the putative consensus binding sequence revealed the exclusion of thymine (uracil in RNA) at base position 4 and a strong inclusion of adenine at base position 5. Interestingly, the predicted SAFB1-binding motif is not dissimilar to purine-rich sequences found in RNA-binding motifs for other SR proteins (reviewed in [26]).

When analysing SAFB1 crosslink sites within protein-coding transcripts, SAFB1 binding density was also enriched in regions encompassing the ORF and 3′ and 5′ UTR. The list of RNA targets was filtered according to the region and density of SAFB1 binding to identify targets that are relevant to tumourigenesis. Several interesting genes were highlighted in this study including* SHF, ITGB4*, and* MALAT-1.*

SHF is a member of a family of adaptor protein characterised by their ability to mediate protein-protein interactions through their Src homology 2 domain [27, 28]. Although the function of SHF is not fully understood, evidence has shown that overexpression of* SHF* significantly decreases the rate of growth factor-induced apoptosis in neuroblastoma cells [27]. Subsequently, Ohira et al. showed that* SHF* mRNA was highly expressed in nonmetastatic neuroblastoma compared to metastatic tumour samples [29]. Another recent study provided evidence that loss of SHF increased cellular mobility and the invasive capability of neuroblastoma cells [30].

Initial iCLIP data from this study revealed enriched SAFB1 binding sites at the alternative promoter of* SHF*. As the aberrant expression of alternative promoters is linked to cancer, SAFB1 binding surrounding this region gathered an interest for further examination. Interestingly, the knockdown of SAFB1 in MCF-7 cells did not significantly alter* SHF* expression while the knockdown of SAFB2 or both SAFB proteins significantly increased* SHF* expression. This suggests that direct SAFB1 binding to the alternative promoter did not affect the expression of this gene. MDA-MB-231 cells were included in this part of the study for comparison and, in this cell type, increased* SHF* expression was observed in the loss of SAFB1 or SAFB2 and both SAFB proteins.

Multiple alternatively spliced transcript variants encoding distinct isoforms have been found for* ITGB4*, although the full function of most variants remains to be defined [31–33]. Alternative splicing mechanism has been indicated to subtly regulate the ligand binding and signalling activity of many integrin subunits (reviewed in [34]). Although the mechanism and significance of alternative splicing in* ITGB4* have not been elucidated, the discovery of SAFB1 binding sites in its exonic regions may provide a new perspective to further understand the mRNA processing of* ITGB4*.

We also identified another potential novel target for SAFB2—*MALAT-1.* Previous work shows that* MALAT-1* colocalises with SRSF2 in nuclear speckles [35]. Furthermore, other splicing factors that localise in nuclear speckles such as SRSF1, SRSF3, and SRSF4 also bind to* MALAT-1* [21, 36]. We, and others [1, 5], observe that SAFB1 distribution has a similar punctate pattern to SRSF2 (Figure 4(d)); it is therefore conceivable that SAFB1 may possess other typical characteristics of a splicing factor which supports its observed function in pre-mRNA splicing [1, 5, 7].

Interestingly, single depletion of either SAFB1 or SAFB2 led to an increase in expression of the other; this pattern was mirrored at both mRNA and protein levels. Our study also suggests that SAFBI and SAFB2 may themselves have different and overlapping RNA targets. This observation supports previous speculations regarding the distinct molecular roles between SAFB1 and SAFB2 [5, 37, 38]. We conclude that SAFB proteins may share multiple similarities in RNA-binding pattern and characteristics with SR proteins. Analysis of SAFB1 crosslink regions and RNA targets confirms previous reports regarding its interaction with other RNA processing machinery and function. Further work will now be undertaken to define whether SAFB1 and SAFB2 function synergistically or compensatory as RNA-binding proteins in breast cancer cells.

## Supplementary Material

Supplementary Figure 1: Generating an iCLIP dataset for SAFB1
Supplementary Figure 2: Distribution of x-link sites and counts in RNAmaps.
Supplementary Figure 3: Compensatory effect of SAFB proteins
Supplementary Figure 4: Distribution of SAFB1 crosslink sites on SHF mRNA.

## References

[1] Nayler O., Strätling W., Bourquin J.-P. (1998). SAF-B protein couples transcription and pre-mRNA splicing to SAR/MAR elements. *Nucleic Acids Research*.

[2] Arao Y., Kuriyama R., Kayama F., Kato S. (2000). A nuclear matrix-associated factor, SAFB-B, interacts with specific isoforms of AUF1/hnRNP D. *Archives of Biochemistry and Biophysics*.

[3] Nikolakaki E., Kohen R., Hartmann A. M., Stamm S., Georgatsou E., Giannakouros T. (2001). Cloning and characterization of an alternatively spliced form of SR protein kinase 1 that interacts specifically with scaffold attachment factor-B. *The Journal of Biological Chemistry*.

[4] Li J., Hawkins I. C., Harvey C. D., Jennings J. L., Link A. J., Patton J. G. (2003). Regulation of alternative splicing by SRrp86 and its interacting proteins. *Molecular and Cellular Biology*.

[5] Sergeant K. A., Bourgeois C. F., Dalgliesh C., Venables J. P., Stevenin J., Elliott D. J. (2007). Alternative RNA splicing complexes containing the scaffold attachment factor SAFB2. *Journal of Cell Science*.

[6] Weighardt F., Cobianchi F., Cartegni L. (1999). A novel hnRNP protein (HAP/SAF-B) enters a subset of hnRNP complexes and relocates in nuclear granules in response to heat shock. *Journal of Cell Science*.

[7] Stoilov P., Dauod R., Nayler O., Stamm S. (2004). Human tra2-beta1 autoregulates its protein concentration by influencing alternative splicing of its pre-mRNA. *Human Molecular Genetics*.

[8] Townson S. M., Kang K., Lee A. V., Oesterreich S. (2004). Structure-function analysis of the estrogen receptor *α* corepressor scaffold attachment factor-B1: identification of a potent transcriptional repression domain. *The Journal of Biological Chemistry*.

[9] Konig J., Zarnack K., Rot G. (2011). iCLIP—transcriptome-wide mapping of protein-RNA interactions with individual nucleotide resolution. *The Journal of Visualized Experiments*.

[10] Acconcia F., Ascenzi P., Bocedi A. (2005). Palmitoylation-dependent estrogen receptor alpha membrane localization: regulation by 17beta-estradiol. *Molecular Biology of the Cell*.

[11] König J., Zarnack K., Rot G. (2010). ICLIP reveals the function of hnRNP particles in splicing at individual nucleotide resolution. *Nature Structural and Molecular Biology*.

[12] Ule J., Jensen K. B., Ruggiu M., Mele A., Ule A., Darnell R. B. (2003). CLIP identifies Nova-regulated RNA networks in the brain. *Science*.

[13] Wang Z., Kayikci M., Briese M. (2010). iCLIP predicts the dual splicing effects of TIA-RNA interactions. *PLoS Biology*.

[14] Ayoubi T. A. Y., van de Ven W. J. M. (1996). Regulation of gene expression by alternative promoters. *The FASEB Journal*.

[15] Davuluri R. V., Suzuki Y., Sugano S., Plass C., Huang T. H.-M. (2008). The functional consequences of alternative promoter use in mammalian genomes. *Trends in Genetics*.

[16] Koch F., Jourquin F., Ferrier P., Andrau J.-C. (2008). Genome-wide RNA polymerase II: not genes only!. *Trends in Biochemical Sciences*.

[17] Singer G. A. C., Wu J., Yan P., Plass C., Huang T. H. M., Davuluri R. V. (2008). Genome-wide analysis of alternative promoters of human genes using a custom promoter tiling array. *BMC Genomics*.

[18] Tripathi V., Ellis J. D., Shen Z. (2010). The nuclear-retained noncoding RNA MALAT1 regulates alternative splicing by modulating SR splicing factor phosphorylation. *Molecular Cell*.

[19] Li L., Feng T., Lian Y., Zhang G., Garen A., Song X. (2009). Role of human noncoding RNAs in the control of tumorigenesis. *Proceedings of the National Academy of Sciences of the United States of America*.

[20] Perez D. S., Hoage T. R., Pritchett J. R. (2008). Long, abundantly expressed non-coding transcripts are altered in cancer. *Human Molecular Genetics*.

[21] Änkö M.-L., Müller-McNicoll M., Brandl H. (2012). The RNA-binding landscapes of two SR proteins reveal unique functions and binding to diverse RNA classes. *Genome Biology*.

[22] Mattick J. S., Makunin I. V. (2006). Non-coding RNA. *Human Molecular Genetics*.

[23] Busch H., Reddy R., Rothblum L., Choi Y. C. (1982). SnRNAs, SnRNPs, and RNA processing. *Annual Review of Biochemistry*.

[24] Wahl M. C., Will C. L., Lührmann R. (2009). The spliceosome: design principles of a dynamic RNP machine. *Cell*.

[25] Rappsilber J., Ryder U., Lamond A. I., Mann M. (2002). Large-scale proteomic analysis of the human spliceosome. *Genome Research*.

[26] Long J. C., Caceres J. F. (2009). The SR protein family of splicing factors: master regulators of gene expression. *Biochemical Journal*.

[27] Lindholm C. K., Frantz J. D., Shoelson S. E., Welsh M. (2000). Shf, a Shb-like adapter protein, is involved in PDGF-*α*-receptor regulation of apoptosis. *Biochemical and Biophysical Research Communications*.

[28] Welsh M., Mares J., Karlsson T., Lavergne C., Breant B., Claesson-Welsh L. (1994). Shb is a ubiquitously expressed Src homology 2 protein. *Oncogene*.

[29] Ohira M., Morohashi A., Inuzuka H. (2003). Expression profiling and characterization of 4200 genes cloned from primary neuroblastomas: identification of 305 genes differentially expressed between favorable and unfavorable subsets. *Oncogene*.

[30] Takagi D., Tatsumi Y., Yokochi T. (2013). Novel adaptor protein Shf interacts with ALK receptor and negatively regulates its downstream signals in neuroblastoma. *Cancer Science*.

[31] Clarke A. S., Lotz M. M., Mercurio A. M. (1994). A novel structural variant of the human *β*4 integrin cDNA. *Cell Adhesion and Communication*.

[32] Tamura R. N., Rozzo C., Starr L. (1990). Epithelial integrin *α*6*β*4: complete primary structure of *α*6 and variant forms of *β*4. *Journal of Cell Biology*.

[33] van Leusden M. R., Kuikman I., Sonnenberg A. (1997). The unique cytoplasmic domain of the human integrin variant *β*4E is produced by partial retention of intronic sequences. *Biochemical and Biophysical Research Communications*.

[34] de Melker A. A., Sonnenberg A. (1999). Integrins: alternative splicing as a mechanism to regulate ligand binding and integrin signaling events. *BioEssays*.

[35] Hutchinson J. N., Ensminger A. W., Clemson C. M., Lynch C. R., Lawrence J. B., Chess A. (2007). A screen for nuclear transcripts identifies two linked noncoding RNAs associated with SC35 splicing domains. *BMC Genomics*.

[36] Sanford J. R., Wang X., Mort M. (2009). Splicing factor SFRS1 recognizes a functionally diverse landscape of RNA transcripts. *Genome Research*.

[37] Hammerich-Hille S., Kaipparettu B. A., Tsimelzon A. (2010). SAFB1 mediates repression of immune regulators and apoptotic genes in breast cancer cells. *The Journal of Biological Chemistry*.

[38] Ivanova M., Dobrzycka K. M., Jiang S. (2005). Scaffold attachment factor B1 functions in development, growth, and reproduction. *Molecular and Cellular Biology*.

