# Supplementary material for: Unravelling the RNA-Binding Properties of SAFB Proteins in Breast Cancer Cells
Source: Biomed Res Int. 2015 Jul 26;2015:395816. doi: 10.1155/2015/395816 (PMC4529905; doi:10.1155/2015/395816)

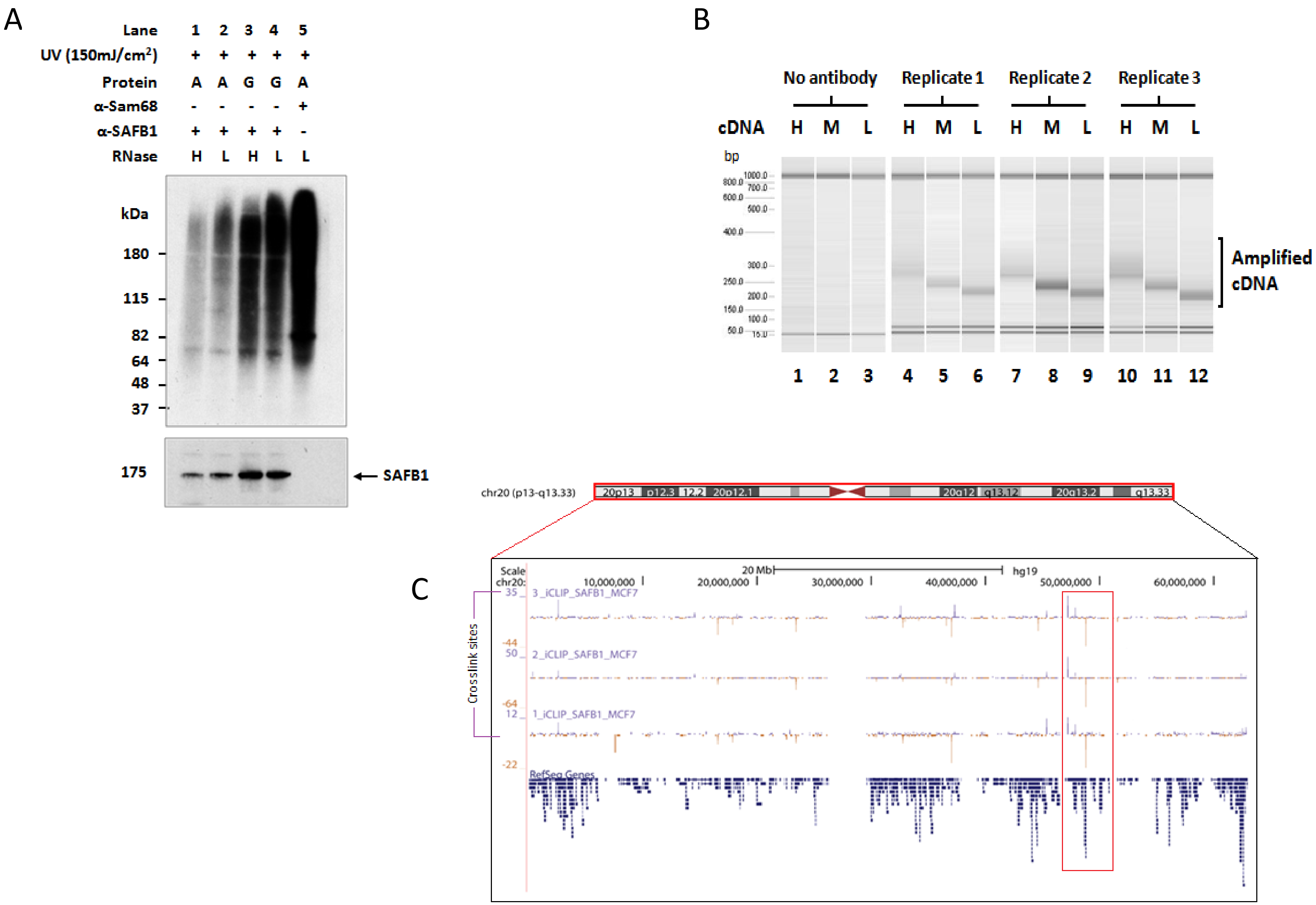

Supplementary Figure 1

A

RNAmap exon-intron

Top 10 junctions with largest x-link sites

position of [junction]	span (upstream; downstream)	associated gene	[x-link sites] (same; anti)
chr2:85133785@-	-173; 300	C2orf89	0; 101
chr5:76129052@+	-269; 287	F2RL1	87; 0
chr15:45490968@-	-165; 300	SHF	68; 0
chr7:139025365@+	-85; 256	C7orf55	66; 1
chr14:102453100@+	-300; 300	DYNC1H1	63; 0
chr11:62296095@-	-300; 300	AHNAK	62; 0
chr1:45241812@+	-253; 174	RPS8	51; 0
chr14:56079289@+	-277; 300	KTN1	46; 0
chr11:62288933@-	-61; 300	AHNAK	43; 0
chr17:38600336@+	-175; 300	IGFBP4	40; 0

B

RNAmap 5-ncRNA

Top 10 junctions with largest x-link sites

position of [junction]	span (upstream; downstream)	associated gene	[x-link sites] (same; anti)
chr11:65269806@+	-1000; 29	MALAT1	220; 0
chr14:20811565@-	-1000; 166	RNaseP_nuc-ENSG00000252678	146; 1
chr9:35658013@-	-429; 132	RNase_MRP-ENSG00000199916	113; 2
chr10:17276831@-	-1000; 66	RP11-124N14.3	0; 111
chr3:58156362@-	-1000; 70	RP11-456N14.2	0; 103
chr16:3202679@-	-1000; 36	AC108134.5	0; 102
chr2:27273131@-	-1000; 42	AC013472.6	0; 101
chr11:62609280@-	-1000; 95	U2-ENSG00000222328	98; 1
chr6:28864306@+	-1000; 100	HCG14	1; 93
chr8:67025246@-	-1000; 36	AC084082.1	0; 84

Supplementary Figure 2

Supplementary Figure 3

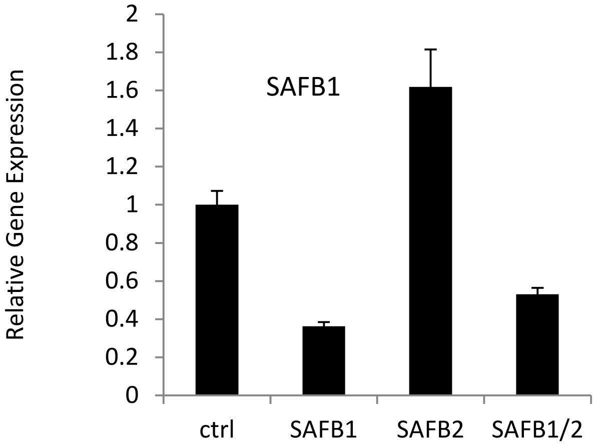

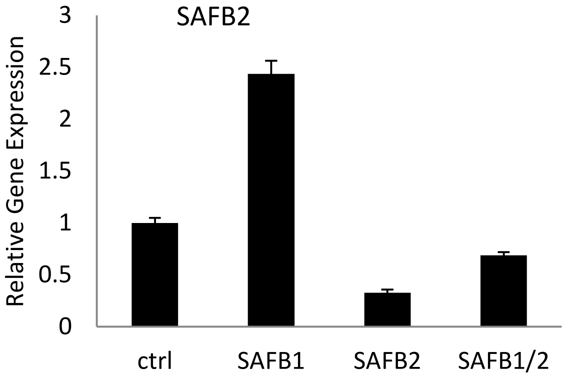

Supplementary Figure 4

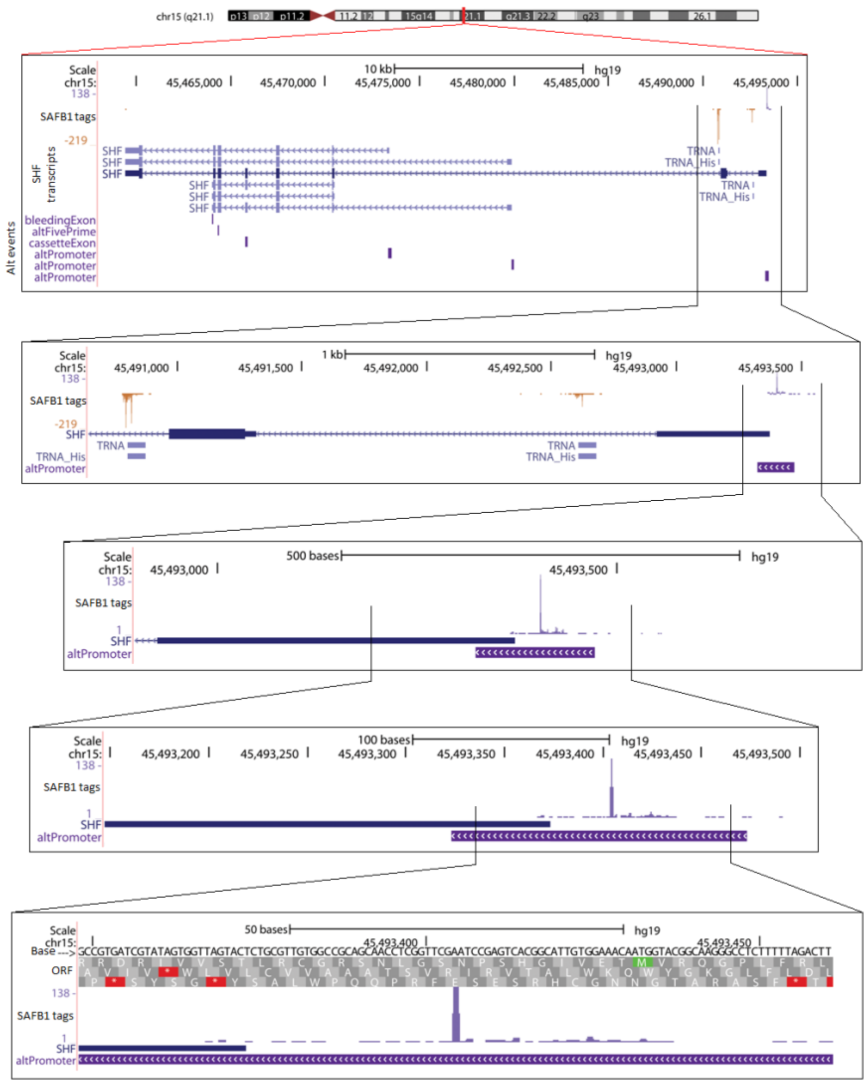

Supplement: Supplementary file 1 — Supplementary Figure 1: Generating an iCLIP dataset for SAFB1 Supplementary Figure 2: Distribution of x-link sites and counts in RNAmaps. Supplementary Figure 3: Compensatory effect of SAFB proteins Supplementary Figure 4: Distribution of SAFB1 crosslink sites on SHF mRNA. [file 395816.f1.pdf]
